# miR-518b Enhances Human Trophoblast Cell Proliferation Through Targeting Rap1b and Activating Ras-MAPK Signal

**DOI:** 10.3389/fendo.2018.00100

**Published:** 2018-03-15

**Authors:** Ming Liu, Yongqing Wang, Huifen Lu, Hao Wang, Xiaoming Shi, Xuan Shao, Yu-xia Li, Yangyu Zhao, Yan-Ling Wang

**Affiliations:** ^1^State Key Laboratory of Stem Cells and Reproductive Biology, Institute of Zoology, Chinese Academy of Sciences, Beijing, China; ^2^Department of Obstetrics and Gynecology, Peking University Third Hospital, Beijing, China; ^3^University of Chinese Academy of Sciences, Beijing, China; ^4^Medical Research Center, Peking University Third Hospital, Beijing, China

**Keywords:** miR-518b, Rap1b, trophoblast, proliferation, preeclampsia

## Abstract

Preeclampsia is a pregnancy-specific complication defined as newly onset gestational hypertension and proteinuria. Deficiency in placental development is considered as the predominant cause of preeclampsia. Our previous study found that the expression of miR-518b increased significantly in the preeclamptic placentas, indicating the potential participation of this small RNA in the occurrence of preeclampsia. In this study, data analysis using multiple databases predicted Rap1b as a candidate target of miR-518b. An evident decrease in Rap1b expression was observed in preeclamptic placentas when compared with the control placentas, which was negatively correlated with the level of miR-518b. Based on the data of *in situ* hybridization and immunohistochemistry showing that Rap1b exhibited similar localization with miR-518b in villous cytotrophoblast cells and column trophoblasts, we further explored their function in regulating trophoblast cell proliferation. In HTR8/SVneo cells, exogenous transfection of miR-518b reduced the expression of Rap1b, and dual-luciferase reporter assay validated Rap1b as the direct target of miR-518b. The small RNA could increase the BrdU incorporation and the ratio of cells at S phase, and enhance the phosphorylation of Raf-1 and ERK1/2. Such growth-promoting effect could be efficiently reversed by Rap1b overexpression. The data indicate that miR-518b can promote trophoblast cell proliferation *via* Rap1b–Ras–MAPK pathway, and the aberrant upregulation of miR-518b in preeclamptic placenta may contribute to the excessive trophoblast proliferation. The study provides new evidence to further understand the etiology of preeclampsia.

## Introduction

Preeclampsia is a complicated pregnancy-associated syndrome featured as newly occurred hypertension and proteinuria after gestational week 20. It affects 3–5% pregnant women all over the world and has been a major cause of maternal and fetal death (1). The pathology of preeclampsia remains unclear, but the deficiencies in placental trophoblast cell behaviors, especially the excessive proliferation of column trophoblasts, shallow invasion into decidua, and insufficient spiral artery remodeling, have been well recognized as the fundamental pathological change in preeclampsia. Accumulating factors are identified to be involved in these cell events, and microRNAs (miRNAs) are suggested as the fine-tune regulatory factors.

MicroRNAs are small non-coding RNAs composed of 19–22 nucleotides. They can bind the 3′UTR of the targeted mRNA and block the translation or accelerate the degeneration of the mRNA, therefore decreasing the protein production (2). The participation of miRNAs in modulating various physiological and pathological processes has been widely demonstrated. Placenta is a miRNA-rich organ, and increasing evidences reveal the important roles of miRNAs in regulating trophoblast cell behaviors. Interestingly, some miRNAs, such as C19MC, C14MC, and miR-371-3 gene cluster, are specifically expressed in the placenta (3), indicating their unique functions in this organ.

C19MC (chromosome 19 microRNA cluster) is the largest primate-specific miRNA cluster. In human genome, it locates in chromosome 19q13.41 with the length of around 100 kb. C19MC can encode 59 mature miRNAs, which are mainly expressed in the placentas, testis, and some tumor cells. The members of C19MC were found to modulate immune-tolerance and viral resistance at the feto-maternal interface (4, 5), and to regulate trophoblast cell differentiation and functions. For instance, miR-512-3p could target PPP3R1 to regulate trophoblast invasion (6). miR-517a/b, miR-517c, and miR-519d could suppress trophoblast cell migration and invasion by targeting a number of migration-related transcripts including CXCL6, FOXL2, NR4A2, and by upregulating the expression of sFlt-1 (7–9). In addition, members of miR-515 subfamily restricted the formation of syncytium *via* suppressing the key genes of trophoblast syncytialization including hCYP19A1, GCM1, and FZD5 (7–10). The abnormally enhanced expression of placental C19MC members was therefore proposed to participate in the etiology of preeclampsia (10–12).

In our previous study, we found miR-518b, a member of C19MC, was significantly upregulated in preeclamptic placentas (13). This small RNA exhibited a gradually increased expression along gestation (14, 15), and its higher circulating level was found in association with gestational hypertension (16). However, its function in placental trophoblast cells remains to be elucidated. Using TargetScanHuman7.1, microRNA.org, miRDB, RNA22v2.0, and TargetMiner database, we found a small G-protein-coupled protein, Rap1b, appeared to be a promising candidate target of miR-518b (17).

In this study, we examined the association of miR-518b and Rap1b in preeclamptic placenta, and further explored the influence of miR-518b on trophoblast cell proliferation by targeting Rap1b. The data provided new evidence showing the involvement of miR-518b in the etiology of preeclampsia.

## Materials and Methods

### Study Participants

The placenta tissues were obtained from the Department of Obstetrics and Gynecology, Peking University Third Hospital, China. The pregnancy outcomes were determined according to the definition in Williams Obstetrics (23rd edition) (18) and the guideline of International Society for the Study of Hypertension in Pregnancy (19). The ethical approval was granted by the Ethic Committees at the Institute of Zoology, Chinese Academy of Sciences (No. IOZ16039) and Peking University Third Hospital (No. 2016-145-03). Written consent was obtained from all of the enrolled individuals. The placentas from severe preeclamptic patients (*n* = 8) and gestational-week-matched preterm labor (PTL) pregnancies without other clinical manifestation (*n* = 8) were collected within 1 h after cesarean deliveries. Specimens at the chorionic plate and basal plate were separately taken from the placenta disk near the position of umbilical cord insertion, and were snap-frozen in liquid nitrogen. The clinical characteristics of the study subjects are shown in Table 1.

**Table 1:** The clinical characteristics of the pregnant women enrolled in this study.

	Preterm labor (*n* = 8)	Preeclampsia (*n* = 8)
Maternal age (years)	28.9 ± 5.5	28.2 ± 4.1
BMI (kg/m^2^)	22.9 ± 3.2	23.3 ± 3.1
Systolic blood pressure (mmHg)	116.9 ± 8.4	155.0 ± 7.6^a^
Diastolic blood pressure (mmHg)	77.5 ± 8.5	99.4 ± 7.3^a^
50-g GCT (mmol/L)	7.1 ± 1.1	6.8 ± 2.3
24-h urine protein (g)	–	3.8 ± 2.1^a^
Gestational day at delivery (day)	233.1 ± 14.9	240.9 ± 22.7
Infant birth weight (g)	2,270 ± 711	2,253 ± 947

*Data are presented as mean ± SD, and significant difference between groups was analyzed by Student’s t-test*.

*^a^Compared with preterm labor, p < 0.05*.

*BMI, body mass index; GCT, glucose challenge test*.

Three human chorionic villi at early gestation (weeks 7–9) were collected at the 306 Hospital of PLA (Beijing, China) from the women who underwent therapeutic termination of pregnancy. No special medical treatment was performed before the termination of pregnancy. The gestational week of chorionic villi was determined according to the morphological observation and pathological examination, with the record of menstrual cycles as a reference.

### *In Situ* Hybridization

Freshly collected tissues were fixed in 4% PFA for 2 h, followed by incubation in serial sucrose solution and embedding in Tissue-Tek O.C.T. compound (Sakura Finetek, Torrance, CA, USA). The sections at 10 µm were fixed in 4% PFA for 15 min, and hybridized with miRCURY LNA miRNA Detection probe labeled with digoxin (RiboBio, Guangzhou, China) at 55°C overnight. After washing in serial saline sodium citrate (SSC) solution, the slides were incubated with AP-conjugated anti-digoxin antibody (Roche, Indianapolis, IN, USA) at 4°C overnight., visualized with BCIP/NBT (Promega, Madison, WI, USA) as substrate, and counterstaining with Nuclear Fast Red (Dingguo Changsheng, Beijing, China). The scramble miRNA labeled with digoxin was used as negative control (NC). The sequences of NC probe were 5′-GTGTAACACGTCTATACGCCCA-3′, and miR-518b probe was 5′-ACCTCTAAAGGGGAGCGCTTTG-3′.

### Immunohistochemistry

Freshly collected tissues were fixed in 4% PFA, dehydrated in serial ethanol, cleared in xylene, and subjected to paraffin embedding. The sections at 5 µm were de-paraffinized in xylene, rehydrated in serial ethanol, and antigen-retrieved in citrate antigen retrieval solution (PH = 6.8) at 95°C for 15 min before being incubated with the primary antibody against Rap1b (SAB2700792, Sigma-Aldrich, Shanghai, China) at 4°C overnight. Incubation with rabbit IgG was used as NC. Following the incubation with HRP-conjugated secondary antibodies (Zhongshan Goldenbridge, Beijing, China) at room temperature for 1 h, the positive signals were visualized with DAB (Zhongshan Goldenbridge) as a substrate. The sections were counterstained with hematoxylin before being mounted.

### Cell Cultures

HTR8/SVneo, an immortalized human trophoblast cell line, was kindly gifted by Dr CH Graham at Queen’s University, Canada (20). The cells were maintained in RPMI 1640 medium supplemented with 10% fetal bovine serum (FBS, Hyclone, Logan City, UT, USA), and passaged every 3 days. Transient transfection of miRNA mimics or plasmids was performed with Lipofectamine2000 reagent (Invitrogen) following the manufacturer’s instruction.

### Protein Extraction and Western Blotting

Tissues or cultured cells were lysed by RIPA buffer containing 1-mmol/L NaF, Na_3_VO_4_, and 1% protease inhibitor cocktail (Sigma Aldrich, St. Louis, MI, USA), and the supernatant was collected after centrifuging at 12,000× *g*. Protein concentration was measured by BCA method (BOSTER, Wuhan, China). The protein extracts were subjected to SDS-PAGE and electronically transferred to nitrocellulose membrane (GE Healthcare, Marlborough, CT, USA). The membrane was blocked with 5% BSA, then incubated with specific primary antibodies which included rabbit anti-Rap1b (SAB2700792, Sigma-Aldrich, Shanghai, China), rabbit anti-p-Raf-1 and Raf-1 (#9421&#9422, Cell Signaling Technology, Shanghai, China), mouse anti-p-ERK1/2, and ERK1/2 (#4376&#4696, Cell Signaling Technology, Shanghai, China) at 4°C overnight. Following incubation with horseradish peroxidase conjugated secondary antibody at room temperature for 2 h (Jackson, MI, USA), the signals were visualized using Super-Signal West Pico Chemiluminescent Substrate (Thermo Scientific, Waltham, MA, USA) and recorded with GeneGnome XRQ (Syngene, Frederick, MD, USA). The images were analyzed with ImageJ software, and the relative density of interest protein was measured by comparing its densitometry values with that of β-actin (DKM9002, Tianjin, China) in the same blot. The stripping buffer (P0025B, Beyotime, Shanghai, China) was used for membrane stripping. Briefly, the membrane was washed in PBST for 5 min, then immersed into the stripping buffer for 10 min. The stripped membrane was washed in PBST for 5 min three times, and blocked with 5% BSA for further detection.

### RNA Extraction and Real-Time PCR

Total RNAs were extracted from the tissues or the cultured cells using TRIzol reagent (Invitrogen) according to the manufacturer’s instruction, and 2-µg total RNA was reverse-transcripted by M-MLV reverse transcriptase into cDNA by oligo (dT) (Promega) or reverse-transcripted into cDNA of miRNA by MiRcute miRNA First-strand cDNA Synthesis Kit (Tiangen Biotech, Beijing, China). The miRNA was reverse-transcribed by adding poly (A) to 3′ end and using oligo (dT)-universal tag as specific primer.

The real-time PCR was carried out according to the instruction of SYBR Premix ER Taq II (Takara, Dalian, China) or miRcute miRNA Premix detection kits (Tiangen Biotech) using LightCycler 480II system (Roche). For the detection of mRNAs, the reaction was carried out at 95°C for 30 s, followed by 40 cycles of 95°C for 5 s and 60°C for 31 s. For measurement of mRNAs, the reaction was carried out at 94°C for 2 min, followed by 40 cycles of 94°C for 20 s, 60°C for 30 s, and 72°C for 30 s. β-actin and U6 were used as internal reference for the interest mRNA and miRNA, respectively. All the amplifications were performed in duplicate. The sequences of primers were as follows:Rap1b, 5′-ATTCCCAGCGTGAGAGGTTC-3′ (forward) and 5′-CTGGCACTGTTGAATTGGGC-3′ (reverse); β-actin, 5′-CGAGCACAGAGCCTCGCCTT-3′ (forward) and 5′-TGCACATGCCGGAGCCGTTG-3′ (reverse); hsa-miR-518b, 5′-CAAAGCGCTCCCCTTTAGAGGT-3′ (forward); hsa-U6, 5′-CGCAAGGATGACACGCAAATTC-3′ (forward). Melting curve was used to detect the specificity of reactions. The fold changes in mRNA and miRNA expression among groups were calculated by the 2^−ΔCq^ method, where ΔCq indicated the subtraction of the Cq of β-actin or U6 from the mRNA or miRNA of interest. The efficiency of the primers of Rap1b, β-actin, miR-518b, and U6 were 0.97, 0.99, 0.95, 0.94, respectively.

### Dual-Luciferase Reporter Assay

The HTR8/SVneo cells in 24-well plate were transfected with 80 ng of pMIR-REPORT plasmid construct, 8 ng of pRL-TK vector as internal reference, and 50 nM of double-stranded miRNA mimics. Forty-eight hours later, the activities of firefly and renilla luciferase were measured using Dual-Glo Luciferase Assay System (Promega) according to the manufacturer’s instructions. The experiments were repeated for three times with triplicate in each group.

### Cell Cycle Assay

The cultured HTR8/SVneo cells were trypsinized at certain time point, and fixed in 75% cold alcohol overnight. The fixed cells were stained by propidium iodide (PI), and subjected to cell cycle analysis with flow cytometer (FACSCallbur, Becton-Dickinson). ModiFit software was used to analyze the ratio of the cells in G0/G1, S, or G2/M phase.

### Cell Apoptosis Assay

Cell apoptosis was measured using Annexin V-FITC Apoptosis Detection Kit (Beyotime, Shanghai, China). In brief, the cultured cells were trypsinized and resuspended in binding buffer. Following stained with FITC-labeled Annexin V and PI, the cells were assayed with flow cytometer (FACSCallbur, Becton-Dickinson). The apoptotic rate was shown as the percentage of Annexin V-positive and Annexin V/PI-positive cells to the total cells.

### Statistics

The statistical analyzes were performed with SPSS 17.0 software (SPSS Inc., Chicago, IL, USA), and the data were presented as mean ± SEM according to the results of at least three repeated experiments. One-sample K–S test was used to detect whether the data followed normal distribution. The comparisons between two groups were performed using Student’s *t*-test, and the comparisons among more than two groups were performed using one-way ANOVA with LSD as the *post hoc* test. *P*-value <0.05 was considered as significant difference.

## Results

### Decreased Rap1b Expression in the Preeclamptic Placentas

**Figure 1:**
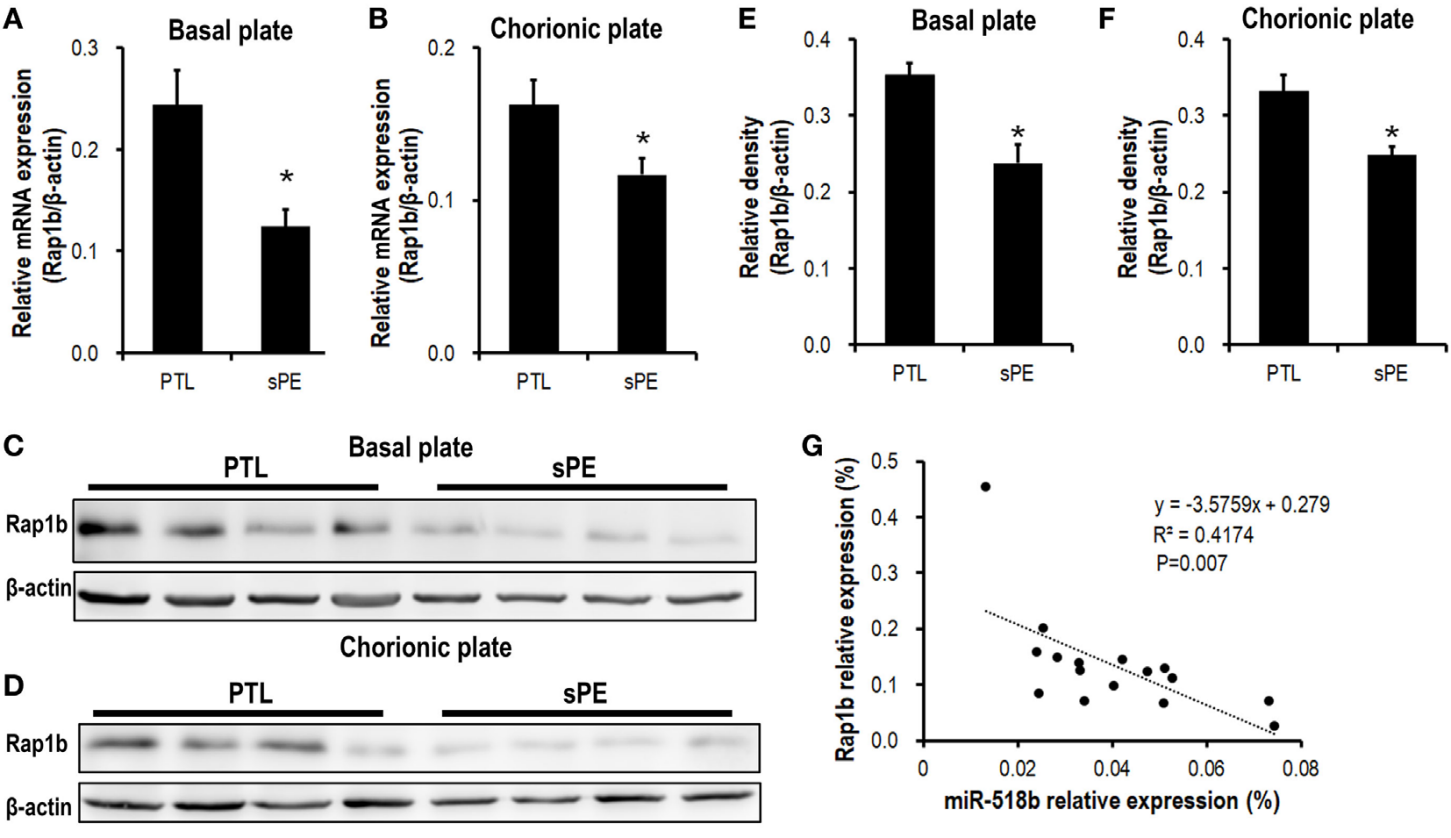
Expression pattern of Rap1b in preeclamptic placentas. **(A,B)** Real-time PCR to measure the mRNA expression of Rap1b in basal plate **(A)** and chorionic plate **(B)** of placentas from severe preeclamptic patients (sPE, *n* = 8) and gestational week-matched preterm labor (PTL, *n* = 8). **(C–F)** Western blotting to measure the protein level of Rap1b in basal plate **(C)** and chorionic plate **(D)** of the placentas from PTL and sPE patients, each of the lanes stood for one patient. **(E)** and **(F)** represent the statistical results of **(C,D)**, respectively. The correlation between the expression of miR-518b and Rap1b in placentas is shown as **(G)**. Data are presented as mean ± SEM. * *P* < 0.05.

We previously identified a significant upregulation of miR-518b in severe preeclamptic placentas (13). Bioinformatic analysis indicated Rap1b as a promising candidate target of miR-518b. Using real-time PCR and Western blotting, we observed an obviously repressed expression of Rap1b in severe preeclamptic placentas (Figure 1). As reported, the gestation-week-matched placentas from PTL patients who had no other clinical manifestation were included as control for preeclamptic placentas (21). In the severe preeclamptic placentas, the mRNA level of Rap1b in basal and chorionic plate decreased to approximately 50 and 70% of corresponding control, respectively (Figures 1A,B). The Rap1b protein level was around 70% of control in both of the basal and chorionic plate of preeclamptic placentas (Figures 1C–F). Using Pearson’s correlation analysis, it was revealed that the placental level of miR-518b and Rap1b was negatively correlated, with *R*^2^ of 0.412 and *P*-value of 0.007 (Figure 1G).

### The Localization of miR-518b and Rap1b in Human Placenta Villi

**Figure 2:**
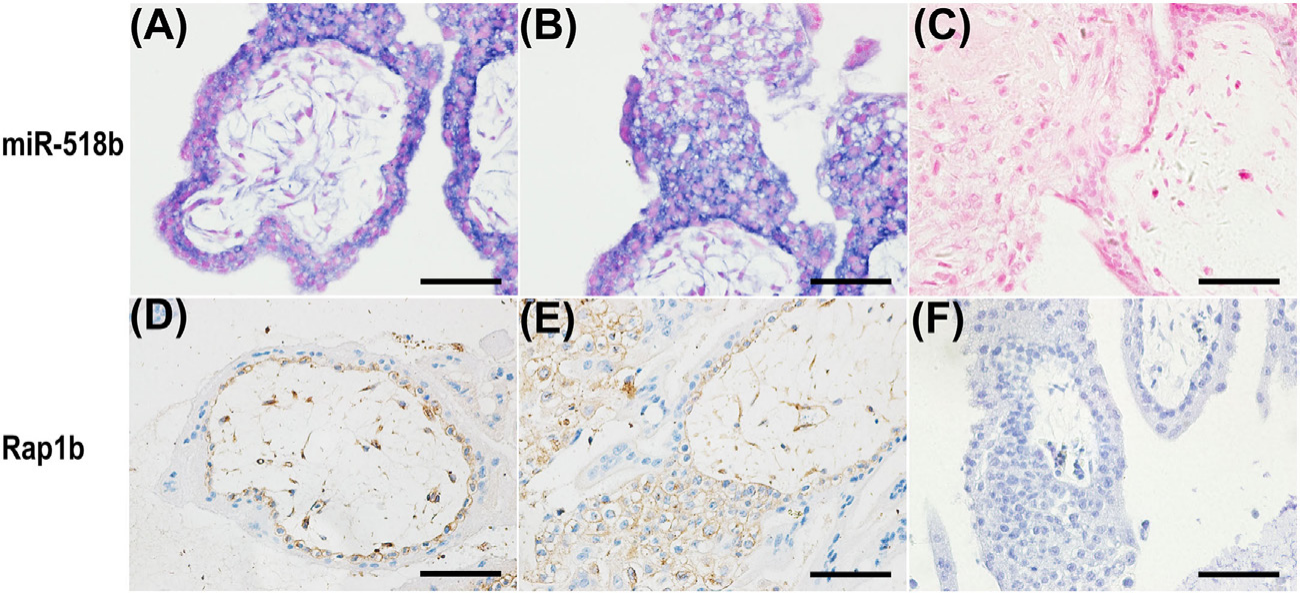
Localization of miR-518b and Rap1b in human placental villi. **(A,B)** The miR-518b expression was measured by *in situ* hybridization (blue) in human placental villi **(A)** and trophoblast cell column **(B)** at gestational weeks 7–9. **(D,E)** The localization of Rap1b was detected by immunohistochemistry in human placental villi **(D)** and trophoblast cell column **(E)**. **(C,F)** showed the negative control of miR-518b (scrambled miR) and Rap1b (rabbit igG). Scale bar represent 50 µm.

*In situ* hybridization and immunohistochemistry were performed to examine the localization of miR-518b and Rap1b in the placenta villi. As shown in Figures 2A–C, miR-518b expressed intensively in cytotrophoblast (CTB) cells, and proximal column trophoblast (PCT) cells, and mildly in syncytiotrophoblasts (STB), and distal column trophoblast (DCT) cells (Figure 2B). The immunoreactivity of Rap1b was primarily observed in CTB cells and PCT cells (Figures 2D–F). Rap1b and miR-518b showed similar expression patterns in CTB and PCT cells, indicating their potential participation in the regulation of trophoblast cell proliferation.

### Validation of Rap1b As the Direct Target of miR-518b in Human Trophoblast Cells

**Figure 3:**
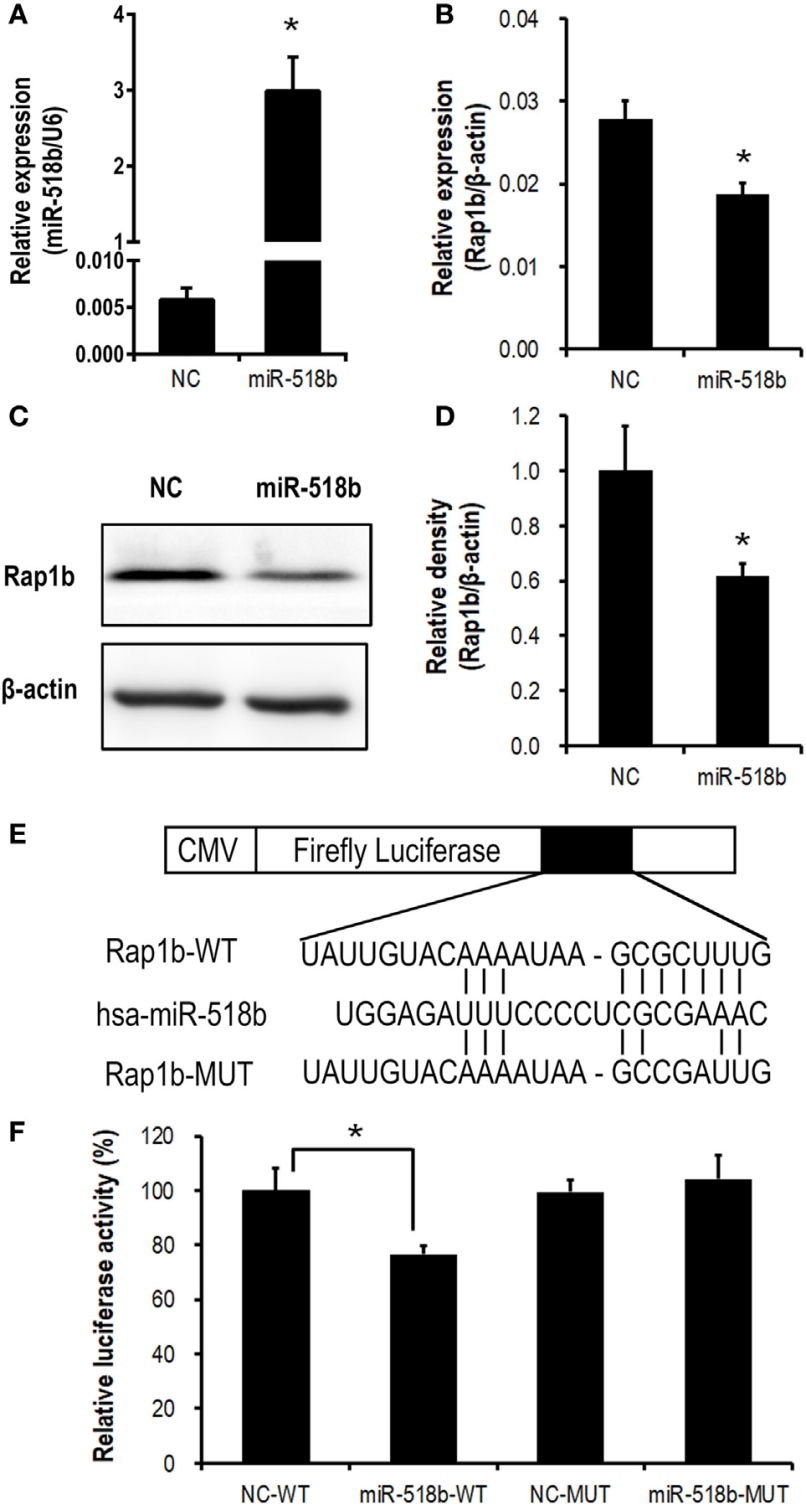
Validation of Rap1b as a direct target of miR-518b in human trophoblast cells. **(A–D)** Change in Rap1b expression upon miR-518b overexpression in human trophoblast cells. The HTR8/SVneo cells were transfected with miR-518b or scramble negative control (NC). The efficiency of transfection was shown in **(A)**, and the expression of Rap1b was revealed by real-time PCR **(B)** and Western blotting **(C,D)**. **(E)** Schematic diagram of the plasmid construction for dual-luciferase report assay. **(F)** Dual-luciferase reporter assay in HTR8/SVneo cells transfected with wild-type (WT) or mutated (MUT) reporter plasmid, and with miR-518b or scramble NC. Each of the tests was replicated for three times. Data are presented as mean ± SEM. * *P* < 0.05.

In the HTR8/Svneo cells that were transfected with miR-518b mimics, the expression of Rap1b was significantly repressed, with the mRNA and protein level reducing to approximately 60–70% of that in the cells that were transfected with scramble control (Figures 3A–D).

Dual-luciferase reporter assay was carried out to further validate the direct targeting on Rap1b by miR-518b. The predicted binding site of miR-518b was localized at 513–519 nt of the 3′ untranslated region (3′ UTR) of Rap1b mRNA. A luciferase reporter plasmid that carried a 170-bp fragment of Rap1b 3′ UTR containing this binding site (Rap1b-WT) or point-mutation in the binding site (Rap1b-MUT) was transfected into the HTR8/SVneo cells, together with miR-518b mimics or scramble control. The luciferase activity in Rap1b-WT group was significantly reduced by miR-518b mimics, while that in Rap1b-MUT group could not be influenced by miR-518b mimics (Figures 3E,F). The data demonstrated that Rap1b was a direct target of miR-518b in human trophoblast cells.

### miR-518b Accelerating the G1/S Entry by Targeting Rap1b in Human Trophoblast Cells

**Figure 4:**
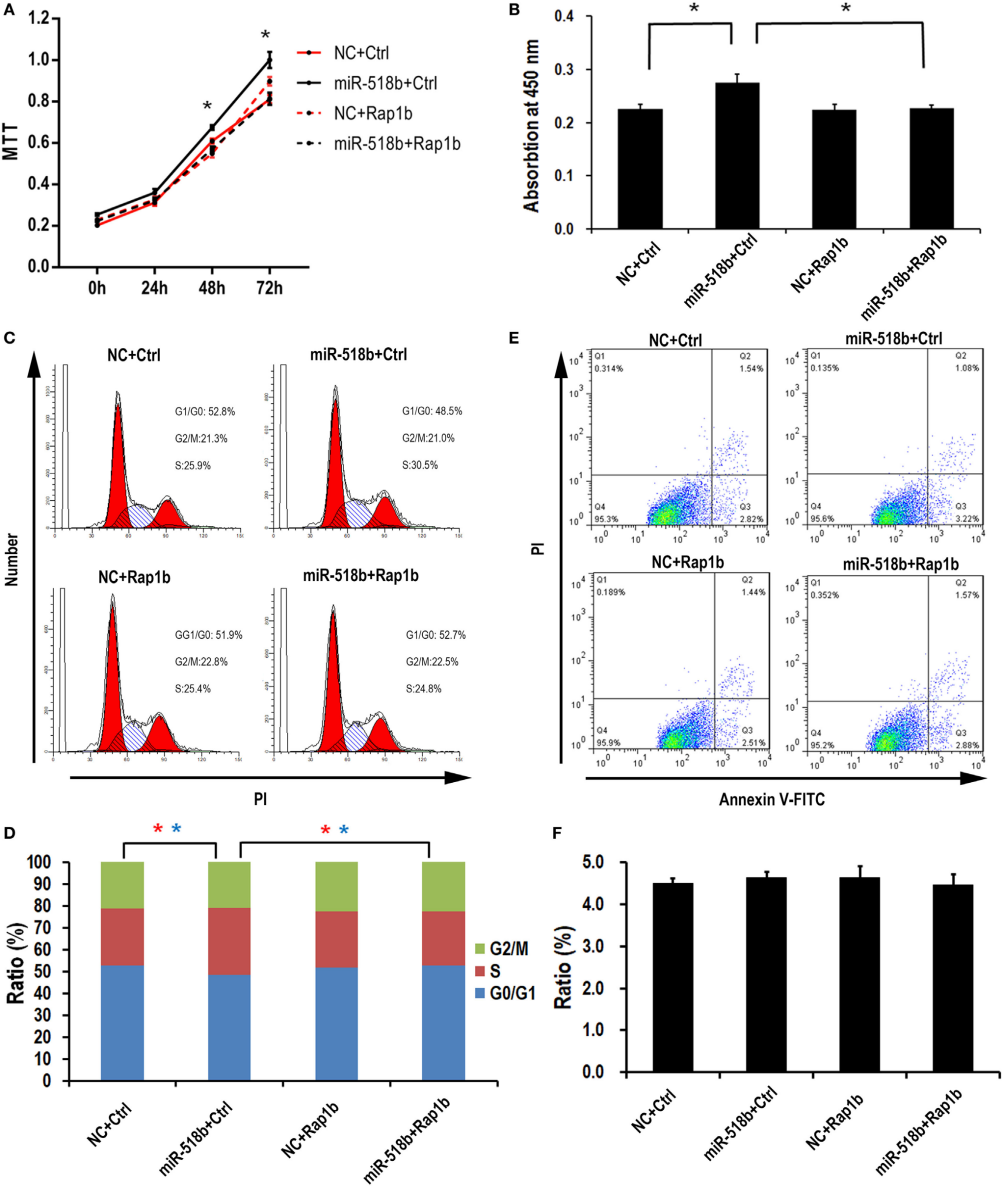
miR-518b transfection accelerates the cell proliferation. **(A)** MTT assay to measure the cell viability in HTR8/SVneo cells. The cells were transfected with miR-518b together with Rap1b overexpression plasmid, and MTT assays were performed at 24, 48, and 72 h after transfection. **(B)** BrdU incorporation assay to measure the cell proliferation in HTR8/SVneo cells transfected with Rap1b overexpression plasmid. **(C,D)** Cell cycle assay for HTR8/SVneo cells transfected with Rap1b overexpression plasmid. **(E,F)** Costaining of propidium iodide (PI) and FITC-Annexin V for cell death/apoptosis assay for HTR8/SVneo cells transfected with Rap1b overexpression plasmid. MTT assay was replicated for five times, and other tests were replicated for three times. Data are presented as mean ± SEM. * *P* < 0.05.

With the MTT assay, it was shown that miR-518b could increase the cell viability 48 h after miR-518b mimics transfection in the HTR8/SVneo cells (Figure 4A). Cotransfection of Rap1b-expressing plasmid and miR-518b could entirely reverse the effect of miR-518b on cell viability (Figure 4A). BrdU incorporation assay showed that the cell growth was enhanced by 30% upon miR-518b transfection, while Rap1b could completely block the growth-enhancing effect of miR-518b (Figure 4B). In addition, the data of flow cytometry revealed that miR-518b mimics significantly increased the ratio of the cells at S phase, and decreased that at G0/G1 phase. Cotransfection of Rap1b plasmid effectively rescued the change in cell cycle caused by miR-518b (Figures 4C,D). Furthermore, Annexin V analysis showed that the cell death/apoptosis were not influenced by either miR-518b or Rap1b (Figures 4E,F). These data demonstrated that miR-518b promoted trophoblast cell proliferation by accelerating the G1/S entry *via* targeting Rap1b.

### The Downstream Pathway of miR-518b and Rap1b Included RAS-MAPK Signaling

**Figure 5:**
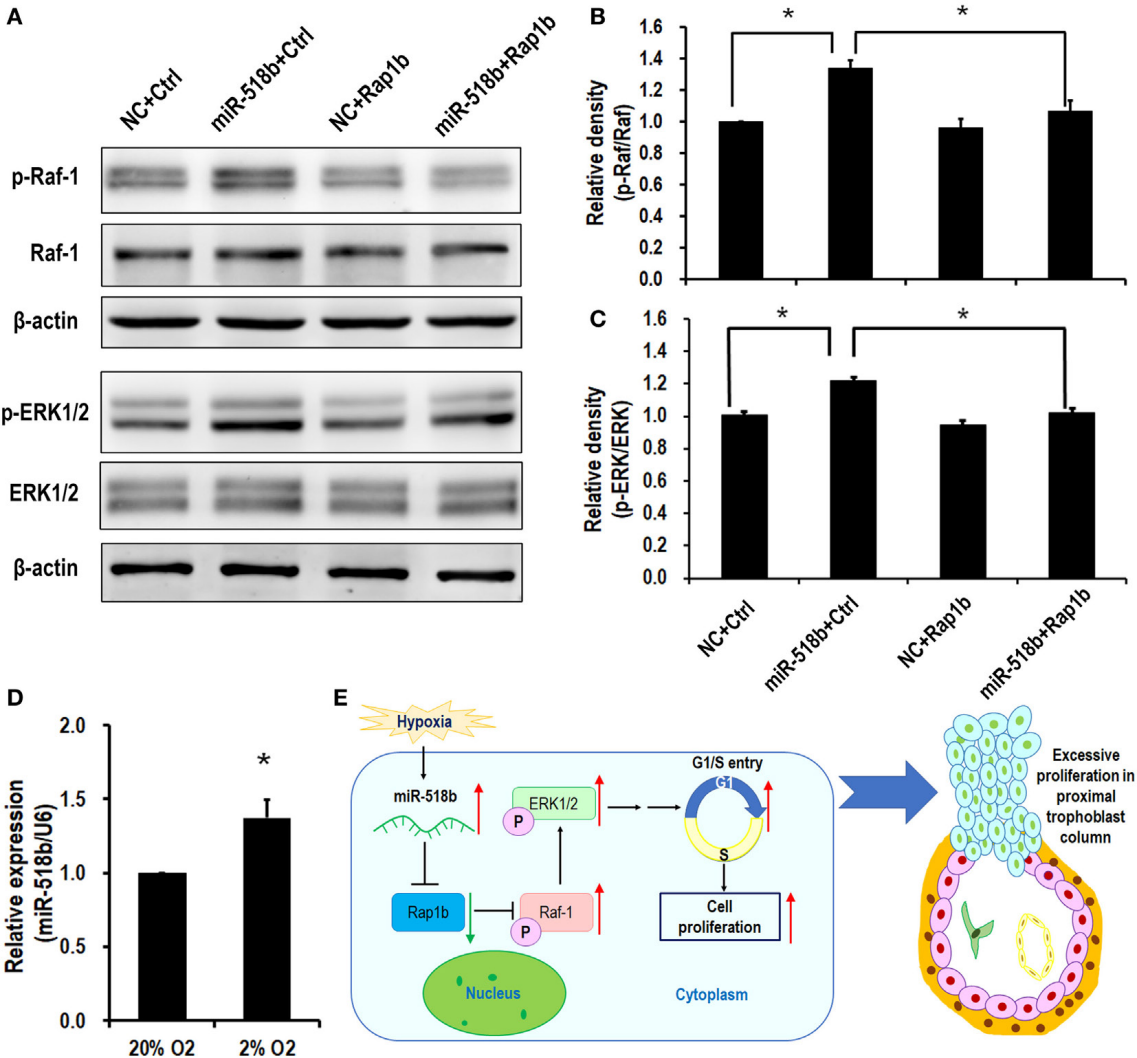
RAS and MAPK are the downstream pathways of miR-518b and Rap1b. **(A–C)** Western blotting to measure the phosphorylation level of Raf-1 and ERK1/2 after transfected with miR-518b or scramble negative control (NC) and Rap1b-encoding plasmid (Rap1b) or control plasmid (Ctrl). **(B,C)** represent the statistical results of p-Raf-1 and p-ERK1/2 shown in **(A)**. **(D)** Change in miR-518b upon hypoxia condition (2% O_2_). Each of the tests was replicated for three times. Data are presented as mean ± SEM. * *P* < 0.05. **(E)** Schematic diagram of miR-518b and Rap1b in trophoblast cell proliferation. miR-518b targets Rap1b, and enhances trophoblast cell proliferation *via* Rap1b–Ras–MAPK pathway. Elevated expression of miR-518b leads to excessive proliferation in proximal column trophoblast cells, indicating that miR-518b may be involved in the etiology of preeclampsia.

Upon transfection of miR-518b mimics in the HTR8/SVneo cells, the phosphorylation level of Raf-1 and ERK increased by approximately 30% compared with the scramble control. To the expectation, cotransfection of the Rap1b-expressing plasmid reversed the increasing in p-Raf-1 and p-ERK that were induced by miR-518b (Figures 5A–C). The data indicated that Ras-MAPK signaling may be involved in the regulation of trophoblast cell proliferation by miR-518b *via* Rap1b. Hypoxia (2% O_2_) induced approximately 37% elevation of miR-518b compared with normoxia (20% O_2_) (Figure 5D).

## Discussion

The present study revealed a new regulator of human trophoblast cell growth. The small RNA, miR-518b, directly targets and represses Rap1b expression, and subsequently hampers the phosphorylation of Raf-1 and ERK1/2, resulting in accelerated G1/S entry and increased cell proliferation. In preeclamptic placenta, the aberrant upregulation in miR-518b expression may therefore cause the excessive trophoblast cell proliferation, especially in column trophoblasts (Figure 5E).

Trophoblast cell proliferation is one of the fundamental aspects for placenta development. In human placenta, villous CTB cells and PCT cells are highly proliferative cells (22), and their growth is intensively regulated by various hormones, growth factors, cytokines, and non-coding RNAs. Pathways involving JAK/STAT, MAPK, PI3K, and Notch2/Notch3, etc., have been reported as regulatory signaling (23, 24). Recent reports demonstrated miRNAs as novel fine-tuning factors for trophoblast cell proliferation. For instance, H19-encoded miR-675 could inhibit trophoblast cell proliferation by influencing Nodal signaling (25). miR-137 reduced trophoblast proliferation by targeting estrogen-related receptor α (ERRα) (26). Knockout of miR-290 cluster decreased the proliferation of trophoblast progenitor cells (27). Members of C19MC have not been reported to modulate trophoblast cell growth, but more likely to influence cell invasion or syncytiolization. Our data of the *in situ* hybridization in this study revealed the predominant expression of miR-518b in proliferative trophoblast cells, and functional study demonstrated its involvement in cell cycle regulation in human trophoblast cells. The results therefore expand our understanding on the members of C19MC in human placental development.

The defects in trophoblast cell proliferation have been reported in various pregnancy complications. In preeclamptic placenta, excessive proliferation of column trophoblast cells was an obvious pathological manifestation (28–30). The cells are considered immature intermediate trophoblast cells, and the aberrantly overgrowing property restricted the cells to differentiate toward invasive phenotype, which lead to the shallow invasion of decidual stroma and spiral arteries by extravillous trophoblasts (30–32). Low-oxygen concentration has been well demonstrated to provoke and maintain the growth property in human placental trophoblast cells (33). The appropriate blood perfusion to the feto-maternal interface will increase the local oxygen tension, and induce the differentiation of trophoblast. The pathological hypoxic condition in the preeclamptic placenta is therefore believed to be causative factor for the abnormal characteristics of the trophoblasts including excessive proliferation and limited invasion. Severe miRNAs have been identified to be responsive for hypoxia, such as miR-210, miR-517a/b, miR-424, miR-365, etc. (7, 34–36). Some of these miRNAs were also revealed to enhance trophoblast cell growth. Here, we found that miR-518b expression could also be induced by low-oxygen tension in trophoblast cells. It is therefore likely that the small RNAs including miR-518b may act as responder for low-oxygen tension to facilitate cell cycle in CTB cells. Their abnormal overexpression may contribute to the pathological properties of the placenta in preeclampsia.

Rap1b is a small GTP-binding protein, which can affect multiple cell behaviors including migration, invasion, and proliferation. The influence of Rap1b on cell proliferation has been controversial. For instance, in sarcoma virus-transformed NIH/3T3, middle T antigen-transformed Rat-2 cells, and keratinocytes, Rap1a/1b were found to inhibit cell proliferation, partially by suppressing Ras pathway (37–39). However, it was also reported that Rap1b activation was required for cAMP-induced G1/S entry, and the phosphorylation of B-Raf was involved (40). In addition, Rap1b knockout mice exhibited proliferation-inhibition in keratinocytes (41). It is most likely that the intracellular microenvironment may determine the effect of Rap1b in cell proliferation. In human placental trophoblast, our data suggested a growth-inhibitory effect of Rap1b through suppressing MAPK activation. The downregulation of Rap1b in preeclamptic placenta may therefore participate in the excessive growth of trophoblast cells.

Rap1b was also found to participate in cell migration and invasion in cancer cells (42). It worked as the downstream of integrin signaling, and accelerated cell invasion *via* remodeling cytoskeletal (43). Integrin switching and signaling activation have been proved as critical ways to control trophoblast cell invasion (44–46). The evidence indicates the role of Rap1b in the regulation of trophoblast invasion. Further investigation in this aspect is needed.

In general, this study demonstrated the regulation of trophoblast cell invasion by miR-518b–Rap1b–Ras–MAPK pathway, and supported a possible role in the development of preeclampsia although further work is required. In-depth investigation on human-placenta-specific C19MC cluster will not only help to understand the regulatory mechanism of human placental development, but also supply potential biomarkers and clinical intervention strategies for human pregnancy-associated complications such as preeclampsia.

## Author Contributions

ML carried out *in situ* hybridization, luciferase assay, cell proliferation assay, data analysis, and manuscript drafting. YW and HL contributed to clinical sample enrollment, data analysis, and interpretation. XS conducted cell cycle analysis. HW and XS performed Western blotting. Y-xL helped to perform cell culture and transfection. YZ and Y-LW contributed to the experimental design, team coordination, data analysis and interpretation, manuscript revising, and submission.

## Conflict of Interest Statement

The authors declare that the research was conducted in the absence of any commercial or financial relationships that could be construed as a potential conflict of interest.
